# The Evolutionary Implications of Hemipenial Morphology of Rattlesnake *Crotalus durissus terrificus* (Laurent, 1768) (Serpentes: Viperidae: Crotalinae)

**DOI:** 10.1371/journal.pone.0066903

**Published:** 2013-06-26

**Authors:** Marcovan Porto, Marco Antonio de Oliveira, Lorenzo Pissinatti, Renata Lopes Rodrigues, Julio Alejandro Rojas-Moscoso, José Carlos Cogo, Konradin Metze, Edson Antunes, César Nahoum, Fabíola Z. Mónica, Gilberto De Nucci

**Affiliations:** 1 Department of Pharmacology, Faculty of Medical Sciences, State University of Campinas, Campinas, São Paulo, Brazil; 2 Institute of Research and Development, University of Vale do Paraíba (UNIVAP) São José dos Campos, São Paulo, Brazil; 3 Department of Pathology, Faculty of Medical Sciences, State University of Campinas, Campinas, São Paulo, Brazil; 4 Department of Pharmacology, Institute of Biomedical Sciences, University of São Paulo (USP), São Paulo, São Paulo, Brazil; McMaster University, Canada

## Abstract

Most amniotes vertebrates have an intromittent organ to deliver semen. The reptile Sphenodon and most birds lost the ancestral penis and developed a cloaca-cloaca mating. Known as hemipenises, the copulatory organ of Squamata shows unique features between the amniotes intromittent organ. They are the only paired intromittent organs across amniotes and are fully inverted and encapsulated in the tail when not in use. The histology and ultrastructure of the hemipenes of *Crotalus durissus* rattlesnake is described as the evolutionary implications of the main features discussed. The organization of hemipenis of *Crotalus durissus terrificus* in two concentric *corpora cavernosa* is similar to other Squamata but differ markedly from the organization of the penis found in crocodilians, testudinata, birds and mammals. Based on the available data, the penis of the ancestral amniotes was made of connective tissue and the incorporation of smooth muscle in the framework of the sinusoids occurred independently in mammals and *Crotalus durissus*. The propulsor action of the muscle retractor penis basalis was confirmed and therefore the named should be changed to musculus hemipenis propulsor.The *retractor penis magnus* found in Squamata has no homology to the retractor penis of mammals, although both are responsible for the retraction of the copulatory organ.

## Introduction

The intromittent organs are functional structures of male organisms with the main function of gamete delivery. They are an adaptation usually observed in organisms that require internal fertilization, and have arisen multiple times in a number of vertebrate and invertebrate lineages [1]. Although the internal fertilization is a synapomorphy to Amniotes vertebrates, most birds and the reptile *Sphenodon* lack intromittent organs and transfer sperm by cloacal apposition [2].

The intromittent organ of Amniotes vertebrate must be an efficient mechanism for transferring spermatozoon to cloaca or vagina of the female [3]. It must be to stiff enough to penetrate the vulva without bending during copulation [1], [3] The *corpora cavernosa* seen arises in amniotes to supply this function, first as a simply hydraulic machine that becomes enlarged by blood. As amniotes form a monophyletic group, we can expect a similar arrange in the penis general morphology. The penile morphology of mammals, which is an integrated part of the complex physiology of male sexual behavioral, varies greatly among the distinct species [4]–[7]. The widely prevalent description of the mammal *corpora cavernosa* is that they are composed of prominent vascular spaces (also known as sinusoidal, lacunar spaces or cave), separated by incomplete fibromuscular trabeculae, rich in smooth muscle [8], [9]. Each hollow shows incomplete fibromuscular trabeculae, rich in smooth muscle. The relaxation and contraction of the smooth muscle controls the erection and detumescence, respectively, of the mammal penis. In birds [2], [10], crocodiles [2], turtles [3], [11], *Lacerta agilis* [12] and some snakes [13]–[15], the *corpora cavernosa* is formed by fibrous elastic connective tissue. Bundles of muscle are generally absent in penis of this group, but was noted in rattlesnake *Crotalus durissus cascavella* [16], in *Crotalus durissus terrificus* [17], in amphisbaenid [18] and *Uroastix hardwickii* lizard [19]. These differences seem to support that penis has evolved independently at least three to four times in amniotes, specifically in mammals, turtles, Squamata, crocodiles, and the Paleognathes and Galloanseridae birds [2], [10].

The morphological variation observed in Reptiles corpora cavernosa strongly reinforces the view of more than one source to penis in amniotes. The genus *Sphenodon* has no male organ for copulation since it has intercourse by cloacal opposition [20]. In turtle and crocodile the penis develops on the ventral cloacal wall [3] and rests within the cloaca when relaxed [2], [3], [11] It is a single medial organ, formed by only one vascular erectile body consisting of a mesh of trabeculae made of connective tissue. The *corpus cavernous* ends distally in a gland similar to what is observed in mammals [11] The Squamata penis, called hemipenis, is the only paired intromittent organ across amniotes [2]. The hemipenises are cylindrical and bilateral structures, that are fully inverted and encapsulated in the tail when not in use [15]. They develop from the lateral walls of the cloaca [21].

In chelonians and crocodiles, the tumescence involves blood alone [3]. The erection is described as sanguineous [2] or as lymphatic and sanguineous in snakes [12], [15]. In birds it is generally considered lymphatic [2], [10].In mammals the erection is a well known blood vascular mechanism [4], [8], [9] Moreover, the hemipenises are functionally independent of one another and only one hemipenis is used during mating, which can last from 3 min to 28 h [22]–[26].

Despite the importance of the intromittent organ to amniotes evolution, even our knowledge on vertebrate penis morphology derives to a great extent from studies performed in humans [4], [9], domestic mammals [5]–[7], [27] and laboratory mammals [28]. Due to its structural characteristics, topography and development, hemipenises stay too far from the pattern observed in other amniotes, and for this reason, it has been suggested that Squamata evolved from reptiles without a penis [1]. However, until the present, the histology of only a few species of Squamata hemipenises been described, with a poor level of detail [1], [12]–[15], [29]. In fact, very little is known about the structure of the tissue that forms the part of the erectile hemipenis and how the sinusoidals are organized to form the *corpora cavernosa*.

In order to understand the relationship between the hemipenis and other Ammonite intromittent organs, a detailed study of the structure and geometry of the *corpora cavernosa* of the rattlesnake *Crotalus durissus terrificus* was performed.

## Materials and Methods

### Animals

All experimental procedures were approved by an Institutional Animal Care and Use Committee (CEUA/UNICAMP: 1655-1 and 2022-1, respectively) and were done in accordance with the Ethical Principles for Animal Research adopted by the Brazilian College for Animal Experimentation. The use of these animals was authorized by the Brazilian Institute of Environment (IBAMA, Sisbio 18020-1). Five adults males of *Crotalus durissus terrificus* (weighing 550±50g) were obtained from Centro de Estudos da Natureza - UNIVAP. The nomenclature tof the general form and superficial ornamentation of the snake hemipenis as had presviouly described [30].

### Light and Scanning Electron Microscopy

Animals were first anesthetized with isoflurane inside a hermetic box, followed by ketamine/xylazine (100 and 70 mg/Kg) injected in different point of ventral side. The hemipenis was exposed by manual pressure on the caudal vein.

For light microscopy, the hemipenises were fixed as above and pieces were embedded in paraffin and sectioned in a rotatory microtome with stainless steel blades. Sections of 4 µm thickness were stained with Masson’s Trichrome, mounted on slides and observed in different magnifications in a Leica DM5000B optical microscope with a digital camera (Leica DFC360 FX, Leica, Germany). The images were stored in TIFF format.

For scanning electron microscopy, the everted hemipenis was fixed by infusion of a fixative solution containing 2.5% of glutaraldehyde diluted in cacodylate buffer 0.1M pH 7.2 for 2 hours. After fixation, sections of 20 µm thick were obtained using a cryostat microtome; the slices were washed in cacodylate buffer 0.1M pH 7.2 and fixed with a solution of 1% osmium tetroxide in cacodylate buffer 0.1M pH 7.2 for 30 minutes. The slices were further washed in cacodylate buffer 3 times and dehydrated in ethanol and dried with hexamethyldisilazane. Dried sections were mounted in aluminum stubs with carbon tape and sputtered with a gold film of 20 nm thickness. The slices were observed in an EVO MA10 (Zeiss) with 15kV accelerating voltage at different magnifications. Digital images were stored in TIFF format.

## Results

**Figure 1:**
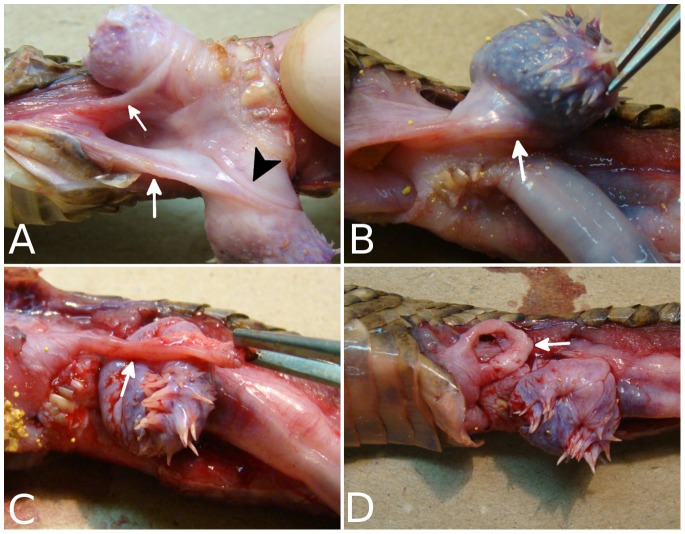
Macroscopic view of the hemipenis of*Crotalus durissus terrificus* partially everted. In A and B it is possible to observed the stretched *m. retractor penis basalis* (arrow) contouring the pedicle of the hemipenis, and participating in *sulcus spermaticus* fold (arrowhead). In C and D it is possible to observes the dissected muscle (arrow) and its loop shape.

The *muscle retractor penis parvus* originates on the cranial caudal vertebrae and inserts a wide aponeurosis on the dorsal asulcate surface of hemipenis (Fig. 1).

The *m. retractor penis basalis* originates from the cloacal shield bellow the integument and inserts via a strong and short tendon on the asulcate side of the hemipenis pedicle. The tendon form a loop that gives a strong knot in the pedicle hemipenes (Fig. 1).

**Figure 2:**
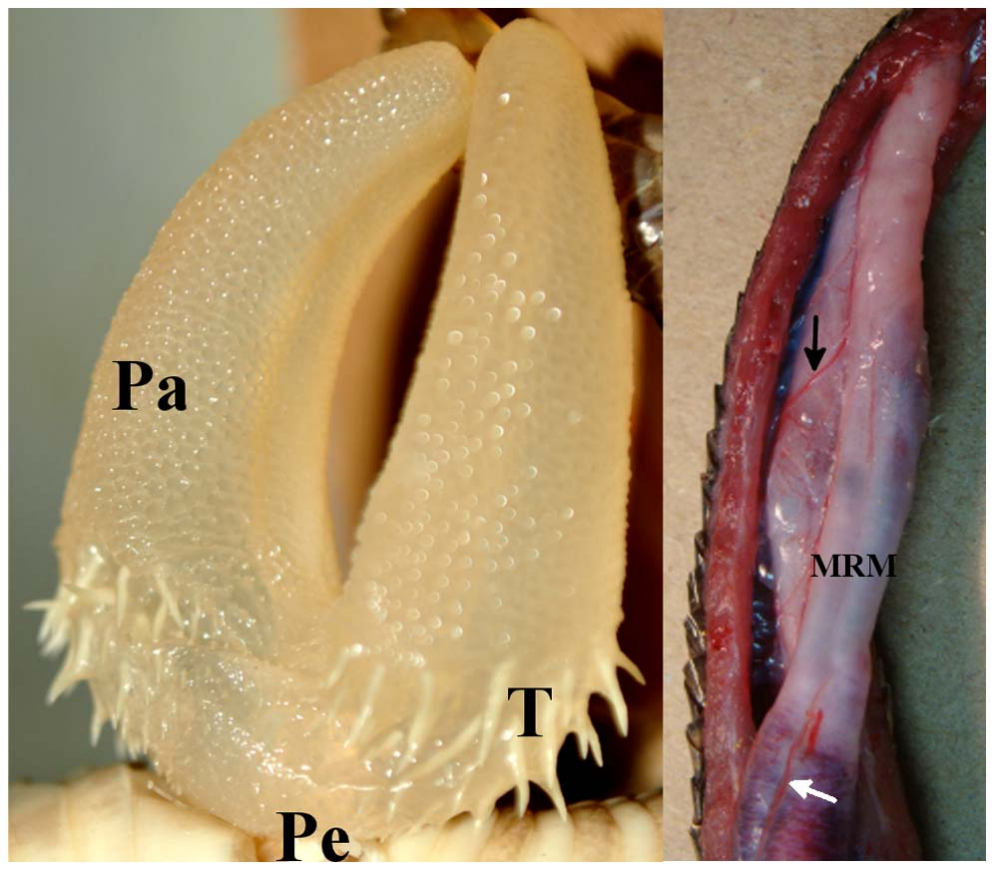
Macroscopic view of the hemipenis of*Crotalus durissus terrificus*. A: Everted hemipenis, B. inverted hemipenis. *musculus retractor penis magnus* (MRM), black arrow points to artery and white arrow points to veins.

Each erected hemipenis of *Crotalus durissus* is a cylinder organ that emerges from the lateral side of cloaca. It is deeply divided, capitate, and covered by large and small spines at the *truncus*. The pedicele is naked. The *capitulum* is covered by papillate cells. The external *sulcus spermaticus* is bifurcated and centrolineal (Fig. 2A).

**Figure 3:**
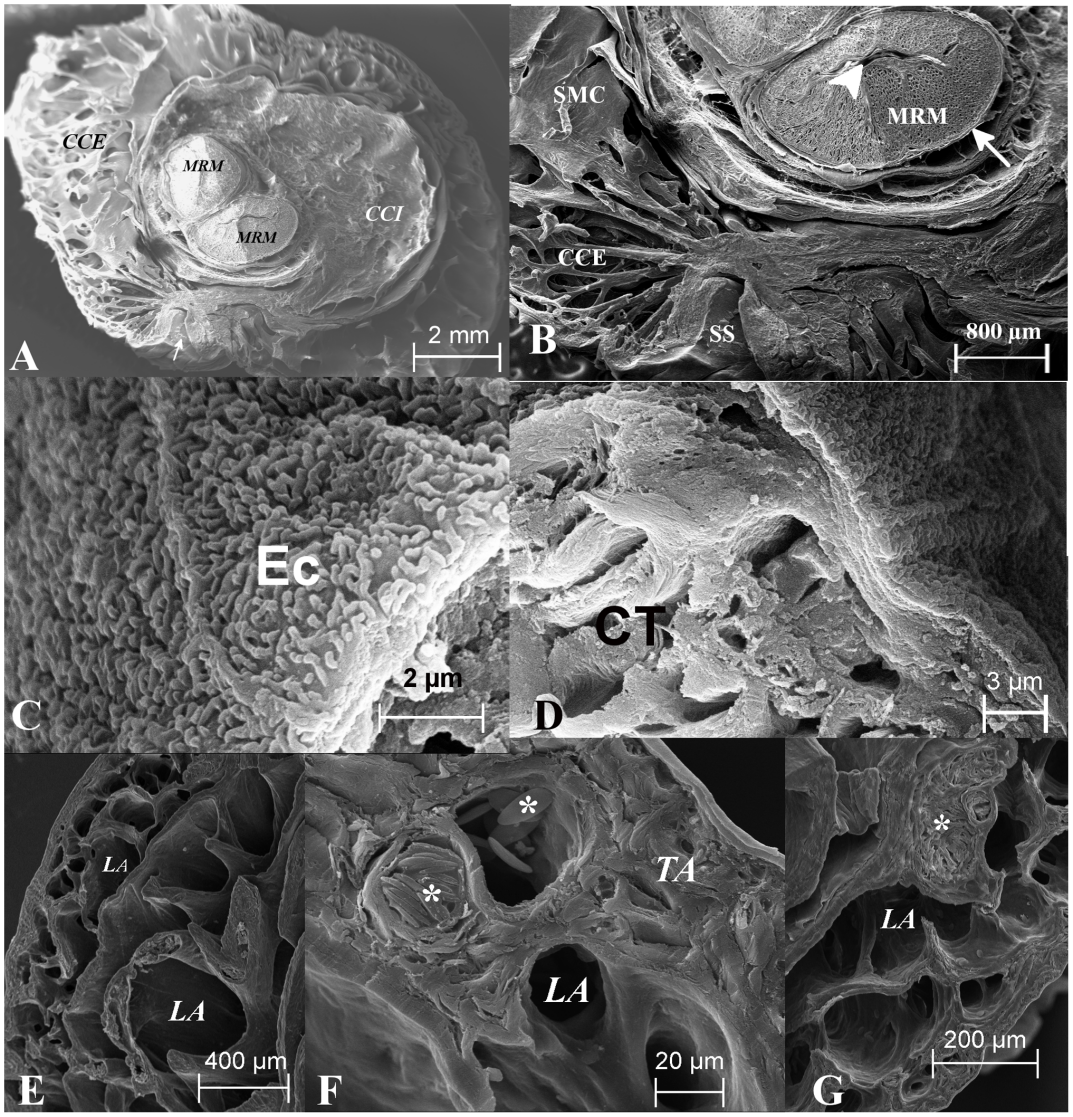
Scanning electron microscopy of the basal region of hemipenis of*Crotalus durissus terrificus*. A: Wide view of cross-section in basal region showing two *corpora cavernosa*, one internal (CCI) and another external (CCE), in central part appear the *musculus retractor penis magnus* (MRM). B: Magnified view of MRM and *sulcus spermaticus* (SS), layers of smooth muscle cells (SMC) and a part of CCE beside SS is observed. C: A view of central part of MRM., showing the central sinusoid (CS) in this muscle and limiting its space there is a tunica albuginea (TA), muscle cells (MC). D: View of MRM where the muscle cells bundles (MC) and the central sinusoid (CS) are observed. E, F and G: Details of sinusoids in external limit of CCE, it is possible to observe a great number of lacuna (LA) some of those full of erythrocytes (*).

**Figure 4:**
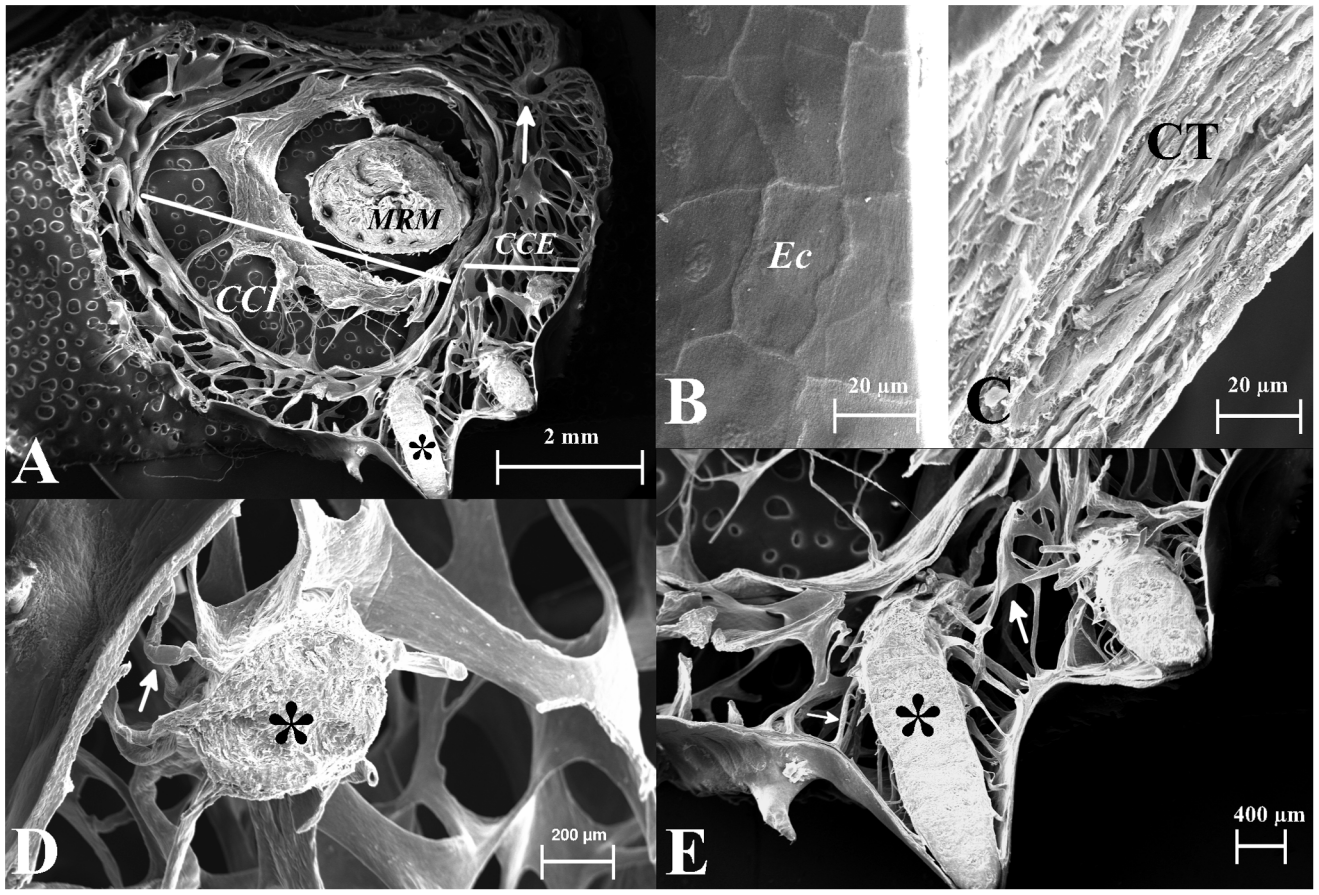
Scanning electron microscopy of the trunk region of hemipenis of*Crotalus durissus terrificus*. A: Wide view of the trunk showing two cavernous bodies, one internal (CCI) and another external (CCE), near the *sulcus spermaticus* (arrowhead); the *musculus retractor penis magnus* (MRM.), spines rounding the trunk (**) and is observed that the number of *trabecula* is greater in CCE than in CCE. B: Detail of external surface of hemipenis in an area without spines, the epithelial cells (EC) covering the *tunica albuginea* (TA) of external face of hemipenis are observed. C: Detail of layers of connective tissue under epithelial cells presented in B. The connective tissue presented is non-cornified and is composed of two-to-five layers. D and E: Detail of insertion point of spine (*) in CCE in the trunk region, arrows point to smooth muscle bundles that anchor the spine in hemipenis.

**Figure 5:**
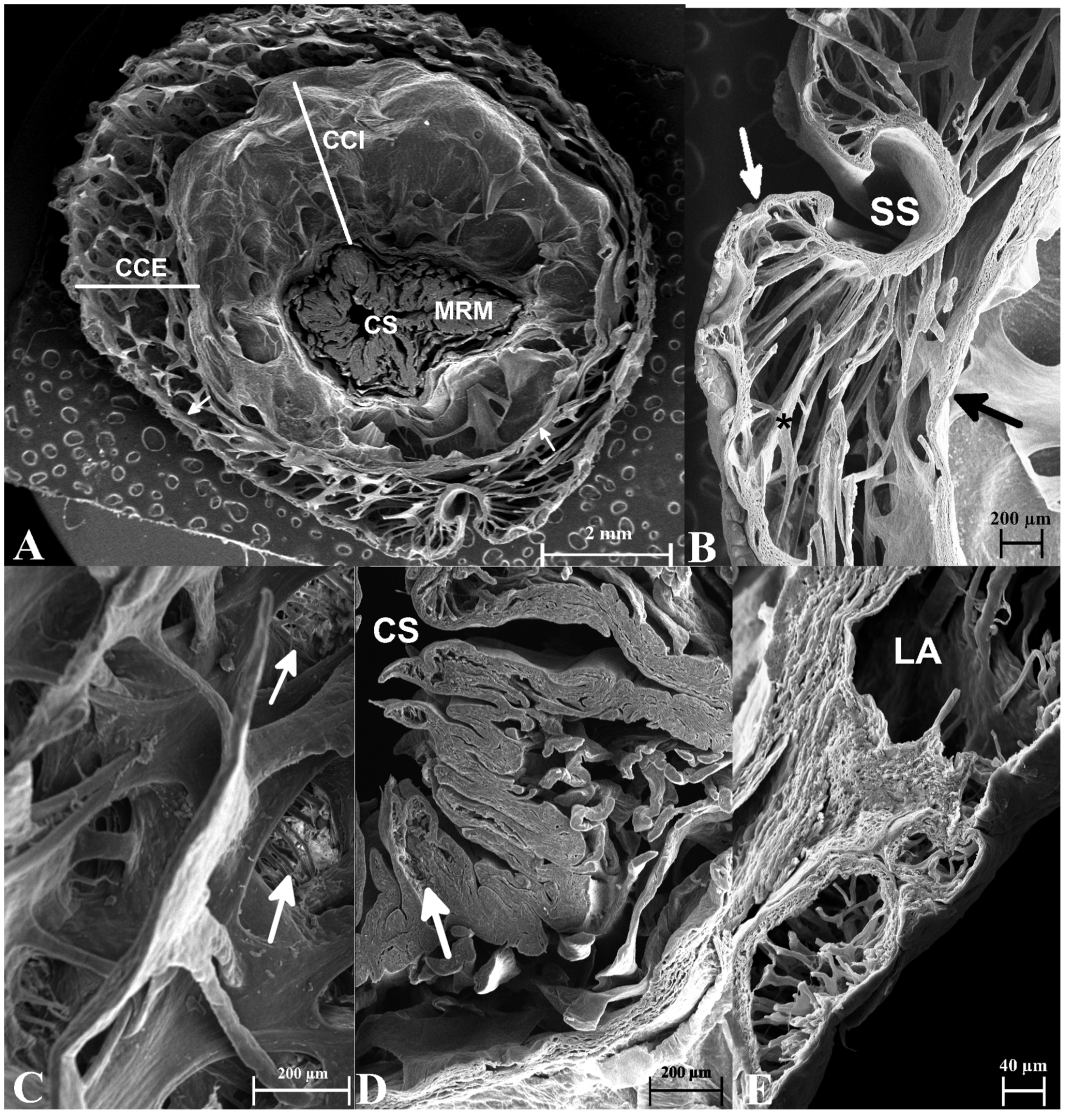
Scanning electron microscopy of the apex region of hemipenis of*Crotalus durissus terrificus*. A: Wide view of the transversal cut from apex region of hemipenis, it is possible to see two cavernous bodies, one internal (CCI) and another external (CCE.), and the *musculus retractor penis magnus* (MRM), arrows point to external and internal sphincters. B: Detail of *sulcus spermaticus* (SS) and CCE, delimited by external (white arrow) and internal (black arrow) sphincters. The *trabeculas* (*) delimit the lacunar space. C: Detail of net formed by smooth muscle bundles (*trabeculas*), arrows point to cluster of erythrocytes rested in bundles. D: Detail of central sinusoid (CS), arrow points to transversal sinusoids inside the muscle. E: Detail of lacunar spaces (LA), with different sizes, presents in external sphincter and in epithelial surface.

The hemipenis consists of two concentric cylinders, named external *corpus cavernous* and internal *corpus cavernous* (Fig. 3A, 4A and 5A).

**Figure 6:**
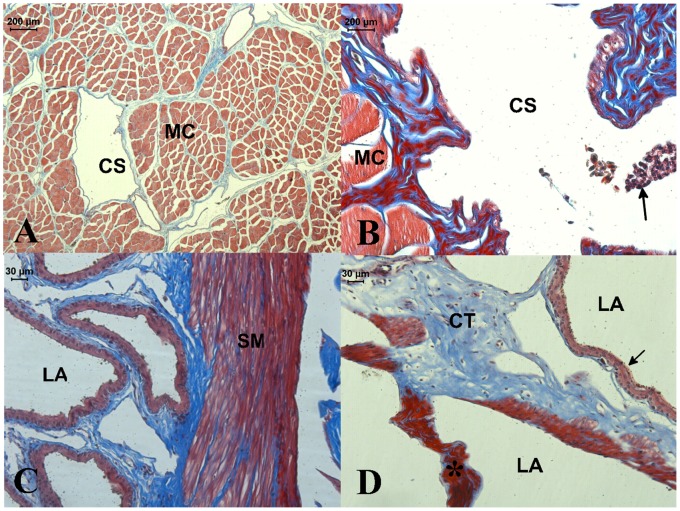
Light microscopy of hemipenis from*Crotalus durissus terrificus.* A: Transversal cut of *musculus retractor penis magnus* showing muscles cells (MC) and the central sinusoid (CS). B: Detail of central sinusoid in a transversal cut of *musculus retractor penis magnus.* The central sinusoid (CS) is layered by smooth muscle cells (MC) and a endothelium, erythrocytes (arrow) are observed inside the sinusoid. C: Detail of *corpus cavernosum* internal, smooth muscle cells (SM); surrounding lacunar spaces (LA). D: Detail of a *trabecula*, the connective tissue (CT) are surrounded by smooth muscle cells (*) and endothelial cells (arrow) forming lacunar spaces (LA).

The hemipenis is coated with epithelial tissue consisting of smooth non-cornified layers composed of two-to-five cells (Fig. 5B and 4C) in the trunk region and coated by papillate cells in the *capitulum*. The epithelial tissue is supported by a dense connective tissue. Small vessels are found in it, but glands are absent (Fig. 4D and 4E). The epithelial tissue limits externally, the external *corpus cavernous*. Internally, the cylinder is limited by a muscular ring made of smooth muscle (Fig. 6C) intermingled with connective tissue and longitudinally oriented (Figure 3A, 4A and 5A).

Throughout the length of the connective tissue there is a mesh of connected and irregular lacunar spaces that become filled with blood during tumescence (Fig and 5C). The presence of these lacunar spaces provides to connective tissue a sinusoidal aspect (Fig. 3 E–G). In the proximal end the bundles of connective tissue are thin and responsible for anchoring the base of the spikes present in the trunk (Fig. 4D and 4E).

The hemipenis is shortened when the muscular rings contract during the detumescence. The muscle relaxation promotes the growth of the organ during the swelling. There are some bundles of smooth muscles crossing the hole of external *corpus cavernous*. They are radially oriented, oblique, and organized in sequence and attached to external and internal rings of smooth muscle. The radial muscles are present throughout the length of the external *corpus cavernous* and act as struts to control the expansion of external *corpus cavernous* during the tumescence of the hemipenis (Fig. 4A, 4E and 6D).

The internal *corpus cavernous* is a hollow cylinder made of loose connective tissue. The external surface is in contact with the inner muscular ring of external *corpus cavernous* (Fig. 5B).

The *m. retractor penis magnus* crosses the *corpus cavernous* internal throughout its entire length and it is the inner wall of *corpus cavernosus* (Fig. 4B). An amorphous sheet of connective tissue lines the caudal end of the internal *corpus cavernous* (Fig. 3A and B), seen with SEM imaging as an irregular sheet in the bottom of the internal *corpus cavernous*.

The *m. retractor penis magnus* arises in the last caudal *vertebras* and enters in internal *corpus cavernous* to insert in the distal end of the hemipenis. It becomes bifurcated as it follows the hemipenis bifurcation (Fig. 3A). Each branch ends at the end of the ipsilateral cranial penile lobe. The *m. retractor penis magnus* is composed by skeletal muscle tissue. It is centrally traversed throughout its length by a narrow sinusoid (Fig. 6A) consisting of a thin layer of smooth muscle lined by endothelium (Fig. 6B).

Some narrow sinusoid, somewhat perpendicular oriented, opens in the central sinusoid and receives blood by arterial branches come from the caudal artery (Fig. 2B). This blood supplies the hemipenis during the tumescence phase of the organ.

Externally, two veins run together near the spermatic groove (Fig. 2B). The veins arise in the internal *corpus cavernous*, at the level of the base of the hemipenis, run cranially and brush off to a venous bed found dorsally and laterally to the cranial region of the hemipenis. The venous bed opens into the caudal vein which runs along side the artery caudal. They are responsible for drainage of blood from the hemipenis during the detumescence.

## Discussion

Beulchelt [29] described the hemipenis erection as result of the blood pressure after the relaxation of the skeletal muscles *retractor penis magnum* and *retractor penis minor retractor penis parvus* and *retractor penis minor*. The actions of this skeletal muscle are responsive by the in-folding of the hemipenis [15]. To evert the hemipenis the lymph and the blood fill the lymphatic sinus cavities, here called *corpora cavernosa*, and at the same time the caudal skeletal muscle, called *m. propulsor penis*, extrudes the hemipenis [15].The first step of mechanical eversion of the hemipenis is due the action of the skeletal muscle named *m. retractor penis basalis* [29]. It rises from cloaca, beneath the skin, to insert in the pedicele of the hemipenis. When the *Crotalus durissus* raises the cloacal shield (opening the cloaca) abruptly, about one-fourth the hemipenis is outfolded by the *muscle retractor penis basalis*. In this phase the hemipenis is not turgid and blood is absent. We have observed the same mechanism in the viperid *Bothrops jararaca* and in the boids *Boa constrictor* and *Epicrates cenchria*. The dissection of the *muscle retractor penis basalis* confirms its propulsor action. Since the action of this muscle is antagonistic to *m. retractor penis magnus* and *m. retractor penis parvus*, the *m. retractor penis basalis* is a misnomer, and should actually be called *musculus hemipenis propulsor.* We have observed that following the partial eversion, the increased hydrodynamic pressure causes the tumescence and eventually the full erection. This second phase is similar to mammals.

The presence of sinusoids in connective tissue underlying the epithelium observed in other snakes has been called lymphatic system or lymph lacunae [13]–[17], [29]. The sinusoid spaces have been observed in lizards and amphisbaenas and were named lymph cisterns or sinus peripheral [12], [18], [31]. Most authors have assumed the exclusive presence of lymph inside sinusoids of connective tissue [12], [13], [15], [29]. However, Rosenberg [18] observed many erythrocytes inside all sinusoids in amphisbaenia.

In birds, the penis tumescence is a lymphatic phenomenon without participation of blood [2], [10]. In this case, a gland resting in the floor of the cloaca supports the phallus with the necessary volume of lymph. Skeletal muscles of the cloaca compress the lymphatic glands to assure the flows of lymph from gland to phallus [2]. In snakes it was postulated that the lymph comes from “cysterna lymphatic” at cloaca [15]. The function of “cysterna lymphatic” or “heart lymphatic” is to draw lymph of the tail and cloacal region [32]. In fact, the lymphatic valves do not allow the reflux of lymph from cysterna lymphatic to the hemipenis. The presence of red blood cells in the sinusoids of connective tissue of *Crotalus durissus* and amphisbaena [18] as well as the absence of a clear mechanism to explain how these spaces could be filled with lymph, allow us to conclude that the erection in Squamata does not involve the participation of lymph, differently from what is observed in birds. Therefore, the name “lymphatic system” or “lymph cisterns” should be avoided, and we use the term “lacunar space” in this paper to avoid misinterpretation.

The paired *retractor penis magnus*, the largest of the hemipenis retractor muscles, originates on a caudal vertebra and inserts on the inside distal end of the corpus cavernous internal of the retracted hemipenis. It is composed by striated muscle bundles. When the hemipenis is everted it is essentially turned “inside-out” and the *retractor penis magnus* muscles extends inside the hemipenis where it is inserted on the inner surface of the free distal end [33]. Until now, the *retractor penis magnus* muscle function was restricted to pull the everted hemipenis back into the snake after eversion [15]. The presence of a bloodstream into the muscle and its role in tumescence of the hemipenis is reported here for the first time. Most mammals have a *retractor penis*, mainly composed of smooth muscle [27]. In mammals, the retractor penis is usually described as being attached to the ventral surface of the first or second coccygeal vertebra, sometimes a sacral attachment is mentioned, and continuing in a caudoventral direction between the *levator ani muscle* and the rectum, to be inserted into the penis towards the end of its free extremity [5]–[7], [27], [34]. This significant difference between snakes and mammals muscle *penis retractor* is best explained by two independent origins for this muscle. There is no homologous structure in reptiles to the retractor penis of mammals.

The smooth muscle architecture and function was confirmed as previously described [16], [17]. Intrinsic muscles are described in *corpora cavernosa* to hemipenis of *Uroastix hardwickii* lizard [19] and amphisbaenas [18] but it is not clear whether the muscle bundles are skeletal or smooth and how they are organized in *corpus cavernous* of these reptiles.

In humans, the bulk of the parenchyma of the *corpus cavernous* consists of bundles of smooth muscle, and the same occurs across the Class Mammalia [9] The state of relaxation or contraction of the smooth muscles is responsible, respectively, for the processes of tumescence or detumescence of the mammalian penis [4]. This condition was observed in the rattlesnake *Crotalus durissus* [17]. However, the structural organization of the smooth muscles fibers is quite different in rattlesnakes and mammals. In mammals the smooth muscles embedded in connective tissue to build the walls of sinusoidal mesh, while in *Crotalus durissus* the smooth muscles fibers are organized in two concentric rings which forming the hollow cylinder called external *corpus cavernous*. Some smooth muscles bundles, radially oriented, are found attached in that rings. This design is unique and must be interpreted as an evolutionary novelty to *Crotalus durissus*.

Intromittent organ made of fibroelastic connective tissue without smooth muscle is found in ratites and Galloanseridae birds [10], crocodiles [2], turtles [3], [11], the lizard *Lacerta agilis* [12] and the hemipenes of the following snakes: *Vipera berus* [13], [29], *Natrix natrix* [13], [29], *Bittis arietans arietans* [14]; this condition was cited to snakes in general by Dowling and Savage [15]. All those reports were based on poor histological material and were not definitive. In fact, the presence of muscles in the framework of *corpora cavernosa* of amphisbaenas [18] and in *Boa constrictor* (personal observation) indicates that this condition could be more generalized in Squamata.

Like other intromittent organ of amniotes, the hemipenises are cylinders flexible enough to enlarge by a hydraulic skeleton filled with fluid before copulation. The hemipenis shares only these similarities with penis of other amniotes. The presence of the following features must be considered as part of a new penis designed, without relationships with other amniotes intromittent organs: two penis housed in the tail, the presence of two *corpora cavernosa* concentrically organized, the absence of true sinusoidal in *corpus cavernous*, the presence of lacunas in connective tissue, and the presence of a skeletal m. retractor penis muscles that conducts blood to hemipenis, besides to retract it. It is interesting that although the hemipenises are a novel structure, the transduction mechanism for erection employed (nitric oxide-cGMP-phosphodiesterase V) is identical to that present in the mammalian penis [17].
